# Is magnetic resonance imaging necessary in isolated greater trochanter fracture? A systemic review and pooled analysis

**DOI:** 10.1186/s12891-015-0857-y

**Published:** 2015-12-24

**Authors:** Seung-Ju Kim, Joonghyun Ahn, Hyung Kook Kim, Jong Hun Kim

**Affiliations:** Department of Orthopedics, KEPCO Medical Foundation, KEPCO Medical center, 308 Uicheon-ro, Dobong-Gu, Seoul, 132-703 Korea; Division of Infectious Diseases, Department of Internal Medicine, Korea University College of Medicine, Seoul, 136-705 Korea

**Keywords:** Greater trochanter, Fracture, MRI

## Abstract

**Background:**

Isolated fractures of the greater trochanter (GT) of the femur are uncommon and few studies have assessed the diagnosis and appropriate therapeutic schedule for these fractures. The current data regarding assessment of isolated fractures of the GT are limited to a few reviews based on the experience of a single institution. Therefore, we asked the following questions: (1) what proportion of cases has an associated extension of the fracture into the intertrochanteric region in isolated GT fracture and (2) what are the treatment options and outcomes of GT fractures with occult intertrochanteric fractures.

**Methods:**

We conducted a systematic review of published studies that evaluated patients who displayed isolated GT fracture on routine radiographic examination and underwent a magnetic resonance imaging (MRI) scan because of the suspicion of extension into the intertrochanteric region. A structured literature review of multiple databases (PubMed, EMBASE, CINAHL, and Cochrane systematic reviews) referenced articles from 1950 to 2015.

**Results:**

A total of 110 patients were identified from 7 published studies. MRI documented isolated GT fractures diagnosed on initial radiographs in only 11 of 110 patients (10 %). In 99 patients (90 %), MRI examinations revealed extension of the fracture into the intertrochanteric region. Surgical fixation was necessary for 61 patients, with a pooled percentage of 55 %. No complications were observed after surgery.

**Conclusions:**

Our study has helped to elucidate further the assessment of isolated fracture of the GT. We believe that MRI is a reasonable option for patients presenting with isolated GT fracture on plain radiographs.

## Background

Isolated fractures of the greater trochanter (GT) are relatively uncommon [1, 2]. Traditionally, the diagnosis of isolated fracture of the GT was confirmed on plain radiographs and non-operative treatment with bed rest was recommended [1, 3]. While a number of studies have described plain radiography diagnosis of simple GT fractures [2–6], it is often difficult to precisely determine the geographic extent of these injuries, and the actual proportion of cases with associated extension of the fracture into the intertrochanteric area is controversial [7–9]. Broader intertrochanteric extension of fractures when standard radiographs show only a GT fracture has been demonstrated [5, 8], and previous studies suggest that magnetic resonance imaging (MRI) has a role in defining the extent of the fracture line in patients who are likely to have a simple GT fracture [5, 10–12]. Lee et al. [2] recommended that all patients presenting with isolated GT fracture on routine radiographs need MRI examination.

Although GT fractures are traditionally managed conservatively when they occur in isolation, a surgical procedure might be necessary if the actual extent of the injury is identified [2, 4]. If the fracture is initially occult, but becomes displaced because of early weight bearing, surgical treatment will be inevitable to correct the deformity and achieve stability; however, this treatment is associated with delayed rehabilitation and even lower long-term survival rates [4, 13, 14]. Nevertheless, consensus is lacking regarding treatment recommendations for isolated fractures of the GT because these fractures are relatively uncommon and the standardized evidence-based guidelines are insufficient. Our current knowledge about the assessment of isolated fracture of the GT is confined to a few reviews based on the experience of a single institution [5, 8]. Further understanding of isolated fractures of the GT would require a large database in order to generate adequate power.

The present study was designed to examine the treatment protocol for isolated fracture of the GT with a review of the literature and pooled analysis. Therefore, we proposed the following questions: (1) what proportion of fractures initially identified as isolated GT fracture actually has extension of the fracture into the intertrochanteric region and (2) what are the treatment options and outcomes of GT fractures with occult intertrochanteric fractures.

## Methods

### Databases searched and search strategy

We performed searches of PubMed, EMBASE, CINAHL, and Cochrane systematic reviews by using the search terms “greater trochanter”, “hip”, “fracture”, “magnetic resonance imaging”, and “MRI”. Two independent reviewers (SJK and JHK) separately performed the search, and each reviewer duplicated the results 2 times. The initial search was performed on January 15, 2015, and it was repeated on March 15, 2015, to ensure accuracy.

### Inclusion and exclusion criteria

The inclusion criteria were as follows: (1) articles published from January 1, 1950 to January 15, 2015, (2) English-written articles in humans, (3) electronic publications that reported cases of GT fracture, (4) both retrospective and prospective series, (5) cases with MRI study, and (6) articles that described a treatment protocol for the fracture.

The exclusion criteria were as follows: (1) patients who had an obvious extension of the fracture line into the trochanteric region on the plain radiographs [2], (2) fractures associated with other occult fractures of the hip (including the acetabulum and pubic rami) [15, 16], (3) articles without data about management and outcome, (4) studies with normal initial radiographs [8, 11, 17], and (5) articles written in a language other than English [18]. We did not limit the number of patients in each study or the minimum duration of follow-up.

**Fig. 1:**
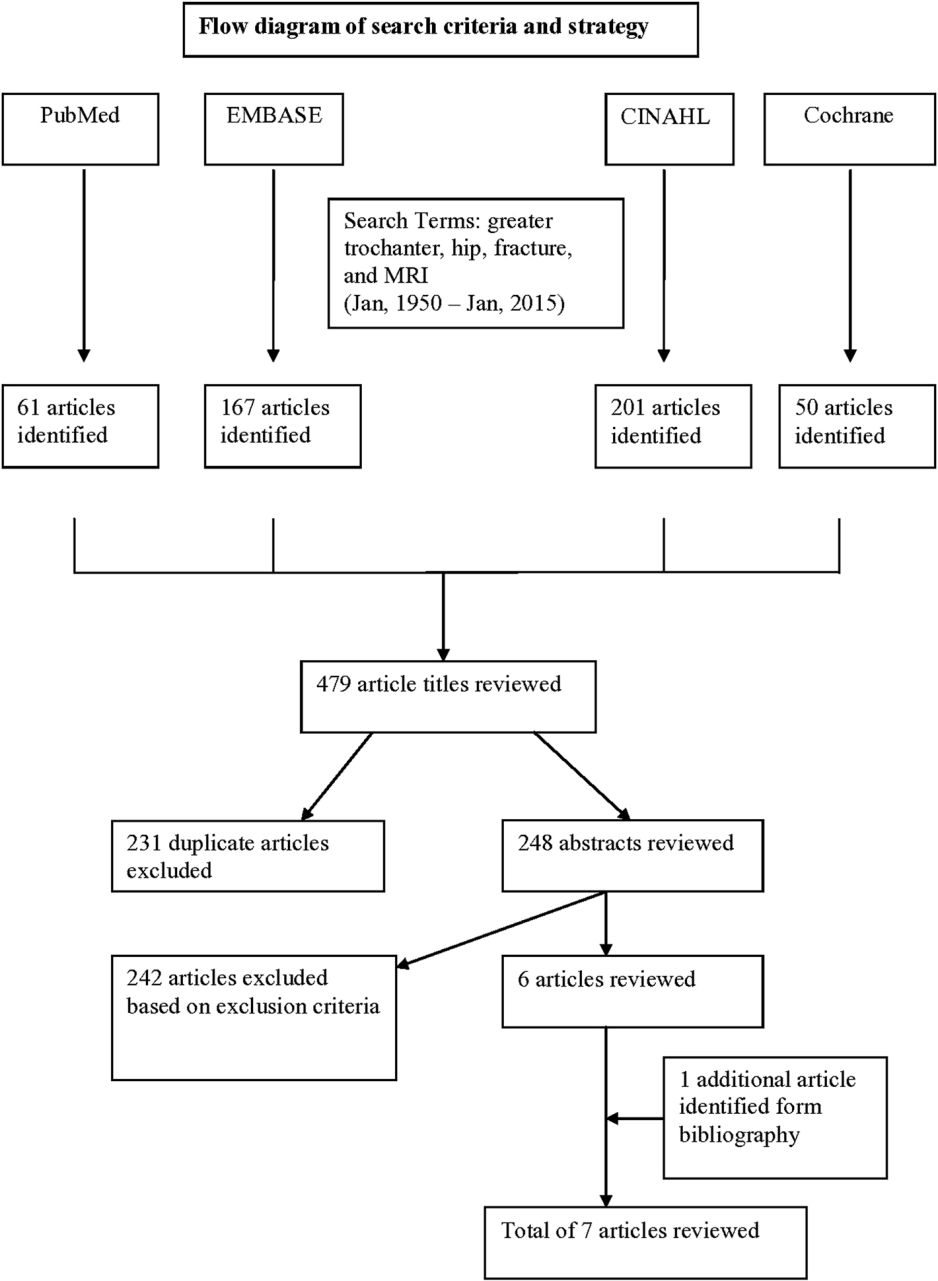
Flow diagram of the search methods and criteria

The first search of the PubMed database yielded 61 articles and the second search of the EMBASE database by using the same search strategy yielded 167 articles. The literature search is summarized in Fig. 1. The search yielded a total of 248 unique articles, with 231 articles appearing in more than 1 of the 4 searches. The study was discussed among the authors if uncertainty existed about inclusion, and a final decision about inclusion was made by consensus. In addition, we screened the references of the obtained articles for any additional studies, and we identified 1 additional article from a bibliography. The full texts of 7 articles were finally obtained and reviewed in detail. Case series were included in our study because of the limited evidence available on the topic. A meta-analysis could not be conducted because of heterogeneity of the reports; the test statistic for evaluating heterogeneity yielded an I^2^ value of 75 % [19]. The preferred reporting items for systematic reviews and meta-analyses (PRISMA) guideline [20] was followed. Studies diagnosing occult intertrochanteric fractures with MRI predominantly started to appear after the report from Schultz et al in 1999 [8], although several prior reports described occult fractures of the hip and proximal femur [11, 17].

### Data extraction and analysis

Two authors (SJK. and JHA) independently extracted the data into Microsoft Excel. The following data were extracted: country of study, demographics including age and gender, initial radiographic findings, time to MRI examination, type of fracture demonstrated by MRI, management of fracture, postoperative rehabilitation, follow-up period, outcomes following surgery, and other complications (displacement of fracture, nonunion, infection, neurological issues, or vascular injury). The study authors were contacted directly for additional clarification if data or information was missing.

## Results

A review of PubMed, EMBASE, CINAHL, and Cochrane literature searches identified a total of 110 patients from 7 selected articles [2, 5, 9, 10, 21–23] that were published from 2000 to 2013. Although complete data were not available, information such as age, gender, and assessment and management of fracture was clearly identified in all of the reports. The mean age of the patients (39 men and 71 women) was 74.3 years and the minimum follow-up period was 3 months (range, 3–31 months). Detailed demographic information is provided in Table 1.

**Table 1:** Demographic information of the studies (NA, not available)

Author	Journal	Year	Study period	Country	N of patients	Male/ Female	Mean age (range)
Lee et al.	Arch Orthop Trauma Surg	2010	July2004–Oct 2008	Korea	25	5/20	72.8 (65–85)
Omura et al.	Arch Orthop Trauma Surg	2000	Jan 1994–Nov 1997	Japan	8	3/5	79.2 (62–101)
Lalonde et al.	Iowa Orthop J.	2010	May 2001–May 2003	Canada	10	5/5	79 (53–90)
Craig et al.	Skeletal Radiol	2000	Mar 1995–Feb 1999	USA	13	10/3	56 (24–86)
Feldman et al.	AJR Am J Roentgenol	2004	1990–2003	USA	37	12/25	NA (50–95)
Kim et al.	J Orthop Trauma	2013	June 2005–Feb 2012	Korea	9	1/8	80.8 (65–91)
Suzuki	Arch Orthop Trauma Surg	2011	Jan 2009–Dec 2010	Japan	8	3/5	78 (58–92)

**Fig. 2:**
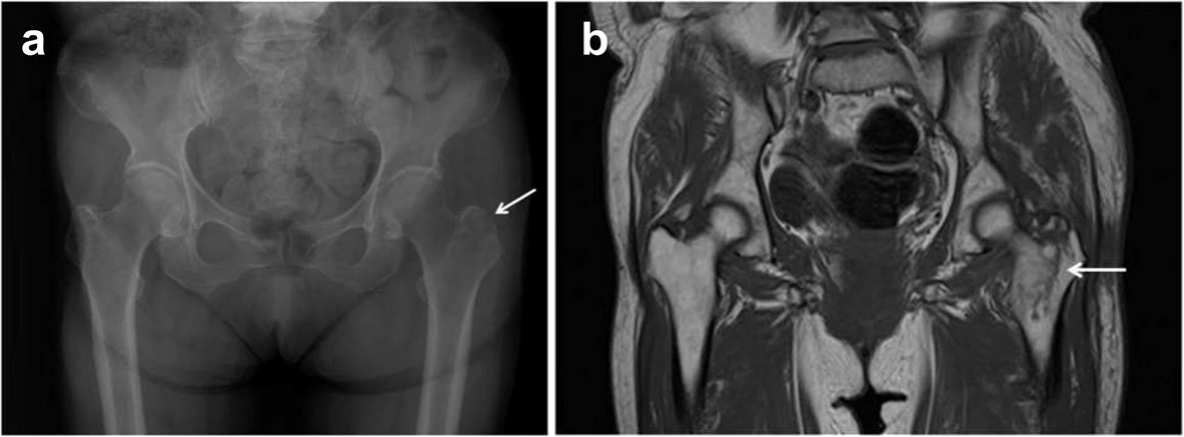
**a** AP radiograph of a 75-year-old woman shows only a minimally displaced isolated fracture of the GT of the Lt hip. **b** MRI reveals a fracture from the GT leading toward the lesser trochanter (more than half way to the medial cortex)

Isolated GT fractures diagnosed on initial radiographs were documented by MRI in only 11 (10 %) of 110 patients (Table 2). In 99 patients, MRI examinations revealed extension of the fracture into the intertrochanteric region, with a pooled percentage of 90 % (Fig. 2); 76 (77 %) of these 99 incomplete intertrochanteric fractures showed extension more than half way to the medial cortex (medial extension beyond the midline on a mid-coronal image). Two patients had intertrochanteric fractures with femoral neck extension [9, 10].

**Table 2:** Data on the studies (NA, not available; GT, greater trochanter; IT, intertrochanteric; Fx, fracture)

Author	Time to MRI	N of GT Fx on MRI	N of IT Fx on MRI	N of Fx crossing midline	N of Surgery	Fixation devices
Lee et al.	Mean 3.9 days (a few hours to 21 days)	5	20	19	15	15 DHS
Omura et al.	Within 5 days	1	7	5	0	No surgery
Lalonde et al.	NA	0	10	0	0	No surgery
Craig et al.	a few hours to 7 days	3	10	6	6	5 DHS, 1 pinning for femoral neck extension
Feldman et al.	3–24 hours	2	35	33	30	NA
Kim et al.	NA	0	9	5	2	2 DHS
Suzuki et al.	Within 7 days	0	8	8	8	5 intramedullary nails, 3 sliding hip hook
Total		11	99	76	61

Surgical fixation was necessary for 61 patients, with a pooled percentage of 55 % (Table 2). The dynamic hip screw (DHS) was the most commonly used device for internal fixation of fractures of the intertrochanteric region [2, 10, 22]. Five proximal femoral nails (3 Stryker Gamma 3 and 2 Zimmer ITST) and 3 sliding hip hooks were also used [23]. Percutaneous pinning was performed for the intertrochanteric fracture with femoral neck extension [10]. No postoperative complications were observed in any of the patients. GT nonunion was noted on imaging studies in 1 patient from the conservative-care group, and the patient experienced residual pain. This patient was treated with open reduction and internal fixation 15 months after the initial trauma [22]. Inconsistencies in reporting made it difficult to analyze the rehabilitation protocols.

## Discussion

MRI has been indicated when plain film radiographs show fracture of the GT of the femur because of the inability of the film to reveal the geographic extent of the lesion, leading to questions about safe treatment [5, 9]. The aim of this report was to determine the proportion of cases with associated extension of GT fracture into the intertrochanteric area and to provide treatment recommendations with a pooled analysis of the reported cases.

In the emergency department, most hip fractures are diagnosed based on a routine radiograph [2, 24]. However, missing the diagnosis of an occult intertrochanteric fracture may lead to fracture displacement, unnecessary surgery, longer hospitalization time, and delayed rehabilitation [2, 23, 25]. Surgical treatment may eventually be necessary for simple GT fracture observed on a plain radiograph for which conservative treatment was initially recommended [4]. Diagnosing the presence or absence of a nondisplaced hip fracture remains difficult, even with high-quality computed tomography (CT) [7]. Although nuclear medicine bone scan is a sensitive examination, fractures may be missed in the first few days after injury with this technique [26]. MRI scans are expensive, but they provide a rapid and anatomically precise diagnosis of hip fracture in patients with normal or equivocal initial radiographs [11]. The literature has focused on the ability of MRI to detect previously overlooked hip fractures [17, 27, 28]. Schultz et al. [8] demonstrated that incomplete intertrochanteric fractures are a distinct subtype of intertrochanteric fracture that can be diagnosed precisely only with MRI. Reiter et al. [4] reported that MRI is the only imaging study that reliably demonstrates the actual complexity of the intertrochanteric extension of a GT fracture, as previous studies showed that both CT and bone scan are imprecise [9, 28]. In our study, MRI examinations revealed extension of the GT fracture into the intertrochanteric region in 99 patients, with a pooled percentage of 90 %. Incomplete intertrochanteric fractures, which were a previously unrecognized phenomenon, are a relatively new MRI-specific diagnosis [15]. We believe that MRI should be used to examine fractures with a radiographic finding of a fissure or fracture of the GT because MRI reveals occult intertrochanteric fracture in most of these cases.

It could be argued that the information delineated in our study is of no clinical importance. The favored treatment for presumed simple GT fractures is most often non-surgical, and early mobilization is allowed because the major weight-bearing portion of the femur is intact [1]. However, our study demonstrates that these fractures can have a broader fracture extending to the intertrochanteric region that cannot be diagnosed by using standard radiographs. Because early weight bearing and motion of the hip joint may cause an incomplete trochanteric fracture to progress to a complete displaced fracture, the decision to manage these fractures conservatively should be carefully considered. The development of a displaced intertrochanteric fracture after a simple non-displaced GT fracture and delayed surgery has been reported recently [4]. Furthermore, in the present study, GT fractures with occult femoral neck extension were identified in several patients [9, 10], and more than half (55 %) of the patients whose initial diagnosis was isolated GT fracture required surgical fixation. Surgical intervention may be required if the actual extent of the injury is known. To date, established guidelines for the treatment of GT fractures with occult intertrochanteric fractures do not exist [2, 21]. Based on previous reports [5, 8, 21, 23], incomplete intertrochanteric fractures that do not cross the midline may be treated non-operatively (1–3 weeks of bed rest, followed by walker-aided ambulation) [2, 5], whereas those that cross the midline tend to be treated surgically. Incomplete intertrochanteric fractures that do not cross the midline on the mid-coronal image are known to be stable, without causing shortening of the lower limb or external rotation deformity on examination [15]. In our study, the DHS was the most commonly used device for internal fixation for fractures that crossed the midline, with a satisfactory success rate and no postoperative complications. Further large multicenter cohort studies are needed to establish objective treatment guidelines for GT fractures with occult intertrochanteric fractures before these recommendations gain widespread acceptance.

### Strengths and limitations of the study

The strength of our study is that we were able to compare many different methods of management and outcomes, and these are the factors that should be considered when deciding whether MRI scanning or surgical fixation is necessary. The main limitation is that the number of studies included in our study was quite small because of our strict exclusion criteria. Therefore, more detailed information that could help to further elucidate the management and outcome of GT fractures with occult intertrochanteric extension was not available.

## Conclusions

In conclusion, patients with a presumed isolated fracture of the GT on standard radiographs may have further fracture extension into the intertrochanteric region. We believe that MRI should be considered for definitive assessment in patients with isolated GT fractures. Although surgical procedures may not be recommended in all of these cases, physicians should be aware that the treatment for this fracture needs to be carefully performed based on clinical experience. Additional prospective studies with a larger number of patients with GT fractures will help to better define the optimal treatment of these injuries and to help improve patient outcomes.
